# Recovery of dried samples from the Helminthological Collection of the Oswaldo Cruz Institute (CHIOC)/Fiocruz through specimen rehydration technique

**DOI:** 10.1590/S1984-29612023021

**Published:** 2023-04-21

**Authors:** Adriana Mainenti, Daniela de Almeida Lopes, Alessandra Gomes da Cruz, Paulo Victor Ramos de Souza, Michelle Cristie Gonçalves da Fonseca, Mônica Ammon Fernandez, Delir Corrêa Gomes, Marcelo Knoff

**Affiliations:** 1 Laboratório de Helmintos Parasitos de Vertebrados, Instituto Oswaldo Cruz, Fundação Oswaldo Cruz, Rio de Janeiro, RJ, Brasil; 2 Laboratório de Malacologia Médica, Instituto Oswaldo Cruz, Fundação Oswaldo Cruz, Rio de Janeiro, RJ, Brasil; 3 Instituto de Tecnologia em Fármacos, Fundação Oswaldo Cruz, Rio de Janeiro, RJ, Brasil

**Keywords:** Collections, helminths, annelids, crustaceans, rehydration techniques, dried specimens, Coleções, helmintos, anelídeos, crustáceos, técnicas de reidratação, espécimes dessecados

## Abstract

The Helminthological Collection of the Oswaldo Cruz Institute is the biggest in Latin America and it is among the largest collections at worldwide reference level, with around 40,000 sets of specimens and approximately one million individual specimens. It contains helminths parasites of vertebrate and invertebrate animals that form part of the fauna of Brazil and other countries. The samples comprise holotypes, paratypes and representative specimens of Platyhelminthes, Acanthocephala, Nematoda and other non-helminth phyla, such as Annelida and Arthropoda. Some of the samples preserved in liquid media were found to have dried out. This made it impossible to analyze these samples morphologically for taxonomic purposes. The aim of this study was to test techniques used for rehydration of the tegument of specimens that had been found to have dried out and present protocols for such techniques. A total of 528 specimens that either no longer were immersed in preservatives or had already dried out were analyzed: 96 digenetic trematodes, 45 cestodes, 22 acanthocephalans, 357 nematodes, four hirudineans and four pentastomid crustaceans. The technique of rehydration using only distilled water on the specimens proved to be efficient for recovering tegument malleability, for all samples analyzed in this present study.

## Resumo

A Coleção Helmintológica do Instituto Oswaldo Cruz é a maior da América Latina e está entre as maiores coleções de referência mundial, com cerca de 40.000 lotes e, aproximadamente, um milhão de espécimes. Seu acervo reúne helmintos parasitos de animais vertebrados e invertebrados da fauna brasileira e de outros países. Seus exemplares são holótipos, parátipos e espécimes representativos de parasitos, pertencentes aos filos Platyhelminthes, Acanthocephala, Nematoda e, ainda alguns espécimes não-helmintos, pertencentes aos filos Annelida e Arthropoda. Parte das amostras preservadas como material líquido foram encontradas dessecadas. Esta condição torna as amostras inviáveis para análise morfológica para propósitos taxonômicos. Portanto, o objetivo deste trabalho foi testar técnicas usadas na reidratação do tegumento dos espécimes que se encontram dessecados e apresentar seus protocolos. Foram analisados 528 lotes, cujos espécimes encontravam-se sem conservantes ou já dessecados: 96 trematodeos digenéticos, 45 cestoides, 22 acantocéfalos, 357 nematoides, quatro hirudíneos e quatro crustáceos pentastomídeos. A técnica de reidratação dos espécimes, utilizando-se apenas água destilada, mostrou-se eficiente na recuperação da maleabilidade tegumentar de todas as amostras trabalhadas no presente estudo.

## Introduction

Over the last hundred years, researchers have deposited significant zoological material in institutional collections in Brazil. Moreover, this material has been highlighted as national and international heritage by several authors since the 1960s (Levi, 1970; Marinoni, 1988; Papavero, 1994; Systematics Agenda, 2000; Lane, 1996). These collections summarize information about species and, when incorporated into efficiently managed computerized databases, they generate greater understanding of the planet's biodiversity. In this context, we include the hundred-year-old Helminthological Collection of the Oswaldo Cruz Institute (CHIOC)/FIOCRUZ, which is the biggest collection of helminths in Latin America and one of the largest collections at worldwide reference level.

CHIOC holds more than 40,000 sets of specimens and approximately one million individual specimens of helminth parasites of animals within the Brazilian fauna in its collection. It represents the biodiversity of the biomes of the Amazon, Atlantic Forest, Cerrado, Caatinga, Pantanal, Pampa, urban areas and continental and marine waters, and the biodiversity of other countries. Its samples comprise holotypes, paratypes and representative specimens of Platyhelminthes (Trematoda, Cestoda and Monogenoidea), Acanthocephala, Nematoda and other non-helminth phyla, such as Annelida (Hirudinea) and Arthropoda (Crustacea) (Knoff et al., 2010; IOC, 2023). All the material is preserved either in liquid media (formaldehyde, acetic formaldehyde, 70% ethanol, 70% ethanol with 5% glycerin or AFA - alcohol, formaldehyde and acetic acid) or as microscope preparations and it is cataloged in files and in a computerized database (Knoff et al., 2010).

In the recent past, some samples of helminth specimens stored in biological collections, museums and universities were found to have accidentally dried out. These were therefore considered to have become inappropriate materials for analysis and research, given that dehydration makes samples brittle and fragile with regard to handling. This brittleness often leads to rupture of the teguments and cuticles of these samples, thus making it difficult or even impossible to observe their external and internal structures.

Nevertheless, there was an awareness that all these stored materials have inestimable value with regard to representation of biodiversity. Hence, some studies on alternatives for recovery of dried-out specimens were conducted. From these studies, techniques aimed at recovering of vertebrate and invertebrate animals of all kinds of phyla were reported (van Cleave & Ross, 1947a, b; Thompson et al., 1966; Kroes, 1980; Schauff, 1986; Vogt, 1991; Huber, 1998; Sanford et al., 2011; Santana & Guimarães, 2014).

Therefore, the purpose of this study was to test the efficiency and usefulness of rehydration methods that had already been used and present a new method for recovering the suppleness and flexibility of dried-out helminth and non-helminth phyla samples deposited in CHIOC, in order to facilitate handling and use of these specimens.

## Material and Methods

**Figure 1:**
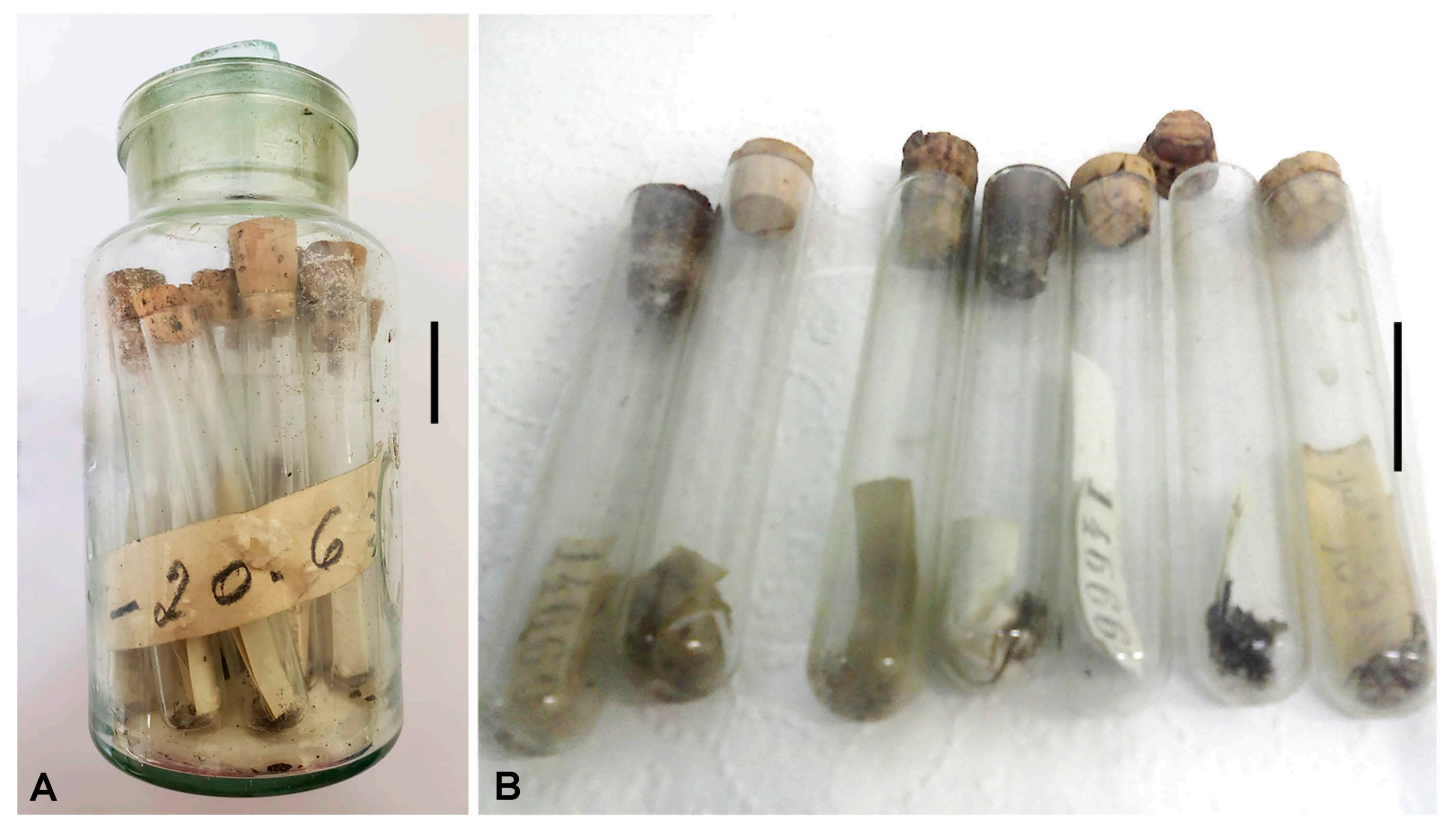
Old samples with dried helminth specimens, deposited in the CHIOC. (A) Vial with test tubes; (B) Test tubes sealed with cork lids. Bars A and B = 20 mm.

Between October 2013 and September 2017, 528 samples of specimens that had dried out and/or only had a little preservative liquid remaining in their storage vials were identified and selected for study. None of these samples were in an appropriate condition for handling. These were old samples that had mainly been deposited in CHIOC between the years 1940 and 1960. They had been kept in test tubes that were sealed with cork stoppers (Figure 1). Unfortunately, this facilitated evaporation of the preserving liquid and darkening of the specimens, due to impregnation of tannin.

These samples were selected from their catalog files, according to their CHIOC collection numbers. They were then were observed and analyzed using a Physis AC stereomicroscope and/or Olympus CX 31 bright-field microscope.

Two rehydration methods were used to recover the specimens: (1) Rehydration using only distilled water; (2) Rehydration using different concentrations of trisodium phosphate (TSP), as described by van Cleave & Ross (1947a). The specimens to be rehydrated were removed from their vials using a fine-bristled brush and were individually transferred to a Petri dish and photographed using a Sony Cybershot camera, model DSC-W30, to record the desiccated state of the sample.

Subsequently, the following procedures were used: (1) 495 samples were immersed in distilled water and observed every 10 minutes, until the ideal rehydration was reached, thus enabling their original morphological characteristics and malleability to be exhibited; these specimens were then transferred to a Petri dish with 70% ethanol, with the exception of nematodes, which were transferred to 70% ethanol with 5% glycerin; (2) 33 samples were tested in distilled water with the addition of different concentrations of TSP (0.25%, 0.35% and 0.5%), in order to make comparisons with the data of van Cleave & Ross (1947a); these samples were immersed in different concentrations and also observed every 10 minutes.

At the end of these procedures, photographs of the rehydrated specimens were taken. After the rehydration process, some darkened specimens were also subjected to rapid immersion in pure glycerin, to enable clearing of the tegument, before they were stored in 70% ethanol, with or without 5% glycerin.

Some samples that consisted of more than one taxonomic group in the same vial were allocated to different new vials and were renumbered. All the rehydrated samples were placed in new glass vials, capped with stoppers and plastic screw caps. They were re-labeled with their deposit numbers, keeping their original information, and their preservative liquid was identified. Sets of specimens with close numbers or in sequence were grouped in single larger flasks, which were filled with 70% ethanol or 70% ethanol plus 5% glycerin (for nematodes) and sealed with plastic films, both inside and outside the screw cap.

The preservation methods used for each group of helminths followed those recommended by Knoff & Gomes (2012). The results from the specimens analyzed were presented in this order: phylum (class, when necessary); optimal rehydration time (minimum and maximum time); size in centimeters (cm); and number of samples.

## Results

Among the 528 dry samples found in CHIOC that were subjected to rehydration tests, 495 samples (approximately 14,000 specimens) were subjected to rehydration in distilled water, and 33 samples (224 specimens) were subjected to different TSP concentrations. Among those subjected to rehydration in distilled water alone, all of them (100%) recovered their malleability and flexibility, thus enabling morphological analysis for taxonomic purposes. The samples submitted to the TSP concentration tests did not reach the ideal rehydration. These rehydration tests were stopped when 50% of the specimens started to present rupture of their tegument or cuticle, even without handling. Among the specimens subjected to immersion in 0.25% TSP (nine samples), 0.35% TSP (11 samples) and 0.50% (13 samples), ruptures in their tissue were observed after 50, 40 and 20 minutes, respectively, and they remained rigid.

The results from analyses on the specimens subjected to rehydration in distilled water that reached an optimal rehydration time, taking in account the different taxonomic groups and sizes, follow below.

### Phylum: Platyhelminthes (Figure 2)

**Figure 2:**
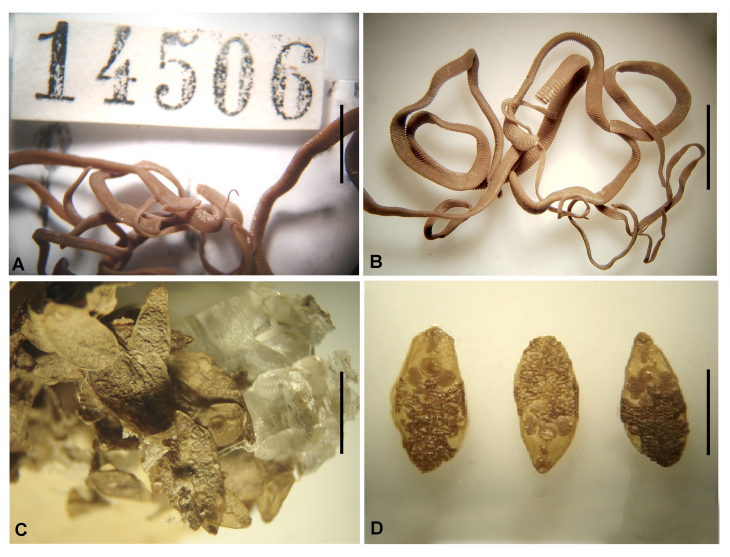
Specimens of Platyhelminthes before and after the rehydration technique. (A) Dried sample of Cestoda not identified (CHIOC 14506), before the rehydration technique; (B) Cestoda (CHIOC 14506) after the rehydration technique; (C) Trematoda, *Platynosomum fastosum* Kossack, 1910 (CHIOC 14107), before the rehydration technique; (D) *P. fastosum* (CHIOC 14107) after the rehydration technique. Bars A and B = 5 mm; C and D = 10 mm.

Class: Cestoda (Figure 2A-2B).

Optimal rehydration time in distilled water: minimum two hours and maximum three hours.

Size: 15 cm to 150 cm.

Number of samples analyzed: 43.

Class: Trematoda (Figure 2C-2D).

Optimal rehydration time in distilled water: minimum two hours and maximum three hours.

Size: 0.2 to 3 cm.

Number of samples analyzed: 82.

### Phylum: Acanthocephala (Figure 3A-3B)

**Figure 3:**
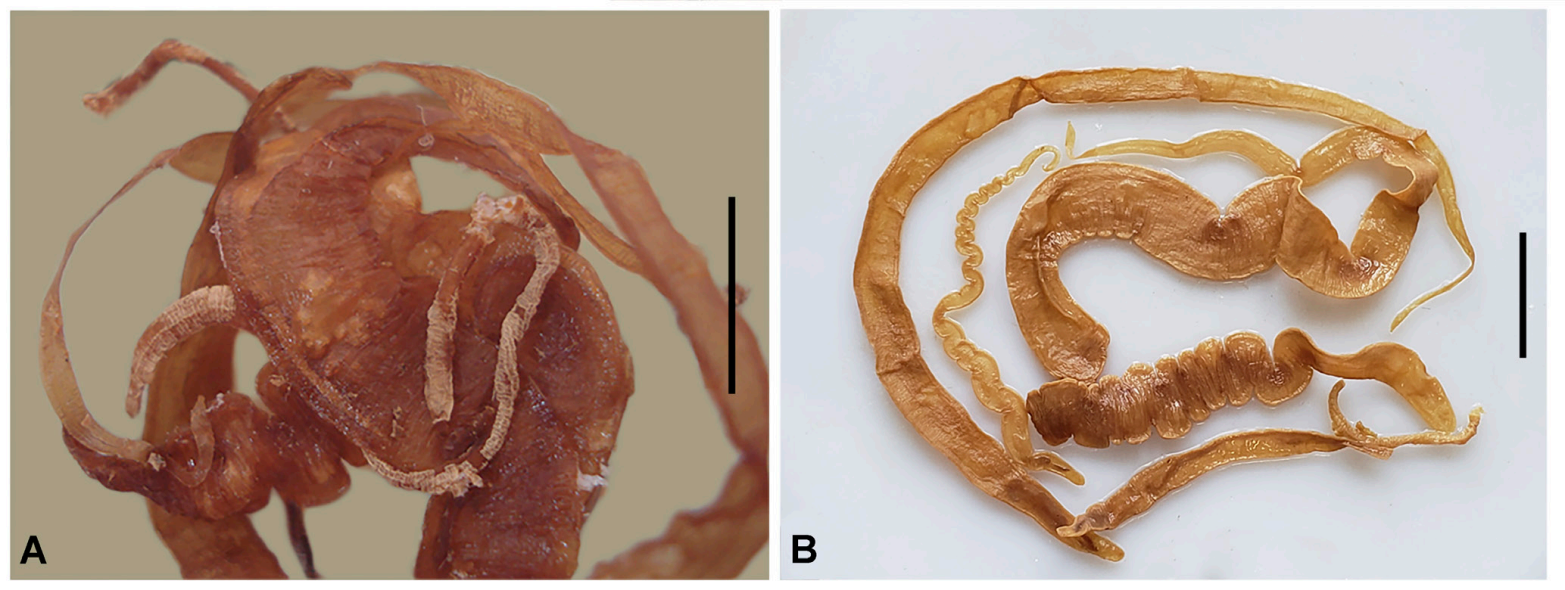
Specimens of Acanthocephala not identified before and after the rehydration technique. (A) Dried sample of Acanthocephala (CHIOC 29512), before the rehydration technique; (B) Acanthocephala (CHIOC 29512) after the rehydration technique. Bars A and B = 10 mm.

Optimal rehydration time in distilled water: minimum four hours and maximum six hours.

Size: 0.5 to 4 cm.

Number of samples analyzed: 22.

### Phylum: Nematoda (Figure 4A-4B)

**Figure 4:**
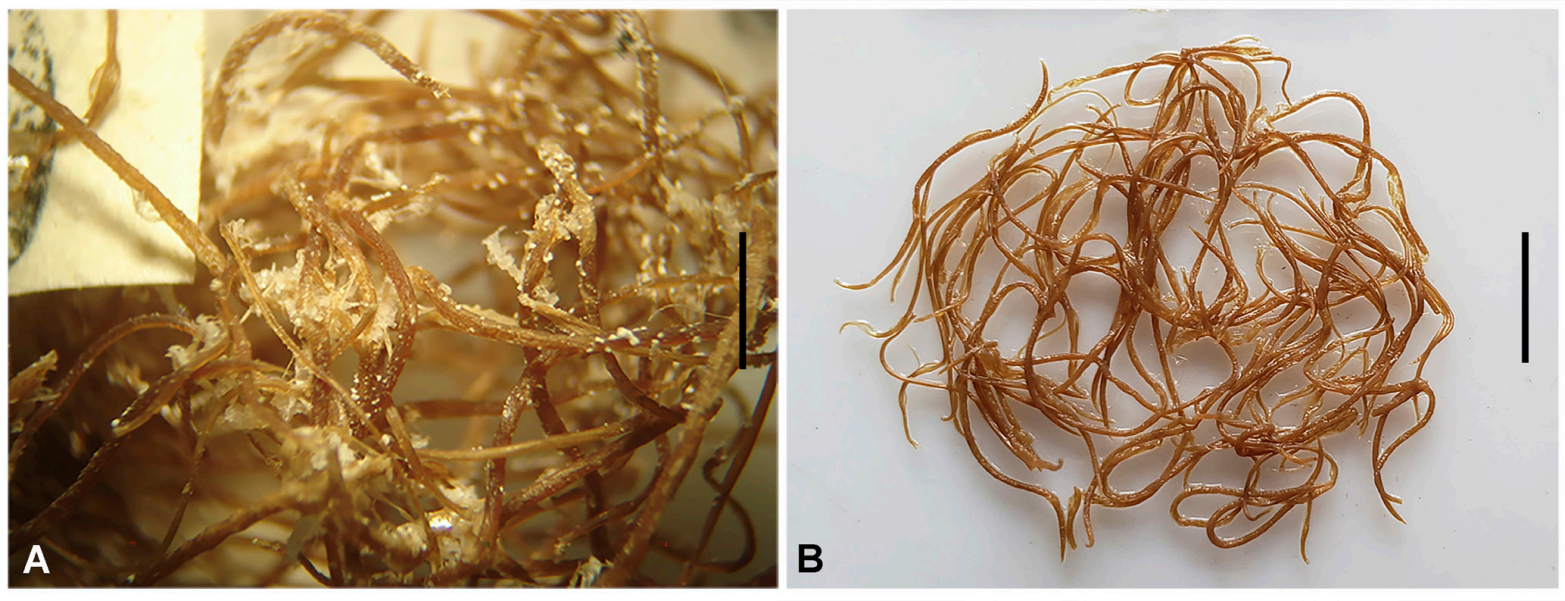
Specimens of Nematoda before and after the rehydration technique. (A) Dried sample of *Bunostomum phlebotomum* (Railliet, 1990) (CHIOC 14359), before the rehydration technique; (B) *B. phlebotomum* (CHIOC 14359) after the rehydration technique. Bars A and B = 5 mm.

Optimal rehydration time in distilled water: minimum one hour and maximum two hours.

Size: 0.1 to 30 cm.

Number of samples analyzed: 341.

### Phylum: Arthropoda (Figure 5)

**Figure 5:**
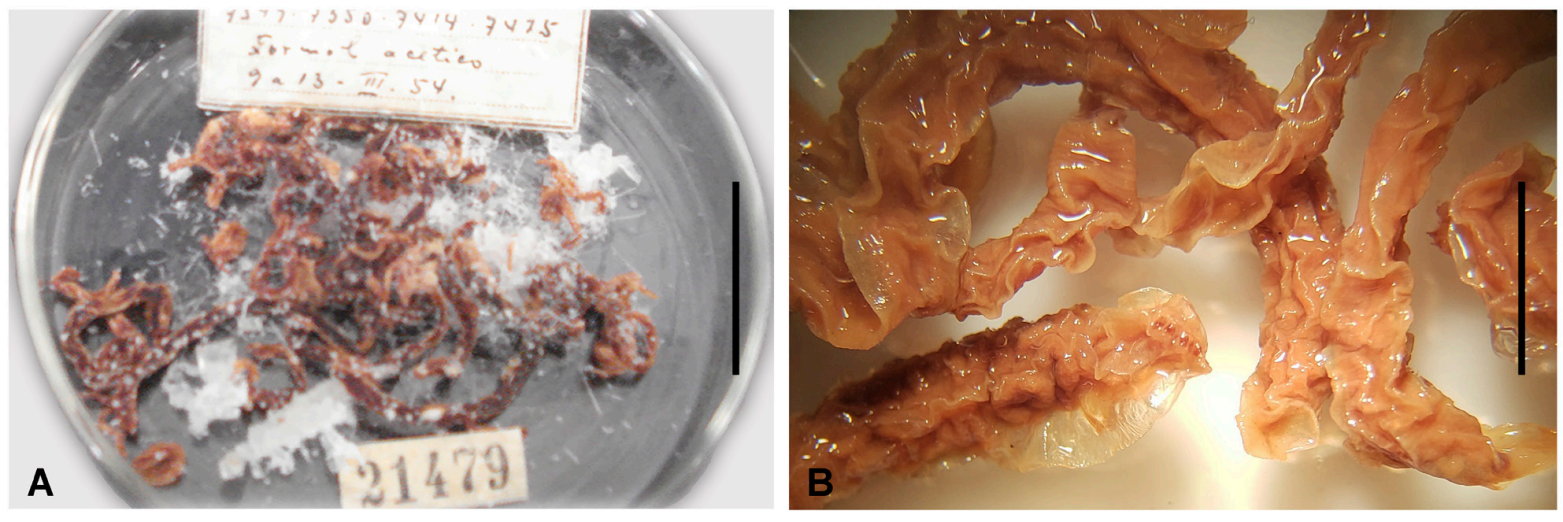
Specimens of Arthropoda before and after the rehydration technique. (A) Dried sample of Linguatulida (Pentastomida, CHIOC 21479), before the rehydration technique; (B) Detail of Linguatulida (CHIOC 21479) after the rehydration technique. Bars A = 20 mm; B = 5 mm.

Class: Pentastomida (Figure 5A-5B).

Optimal rehydration time in distilled water: 24 hours.

Size: 3.5 to 6.5 cm.

Number of samples analyzed: 3.

### Phylum: Annelida (Figure 6)

**Figure 6:**
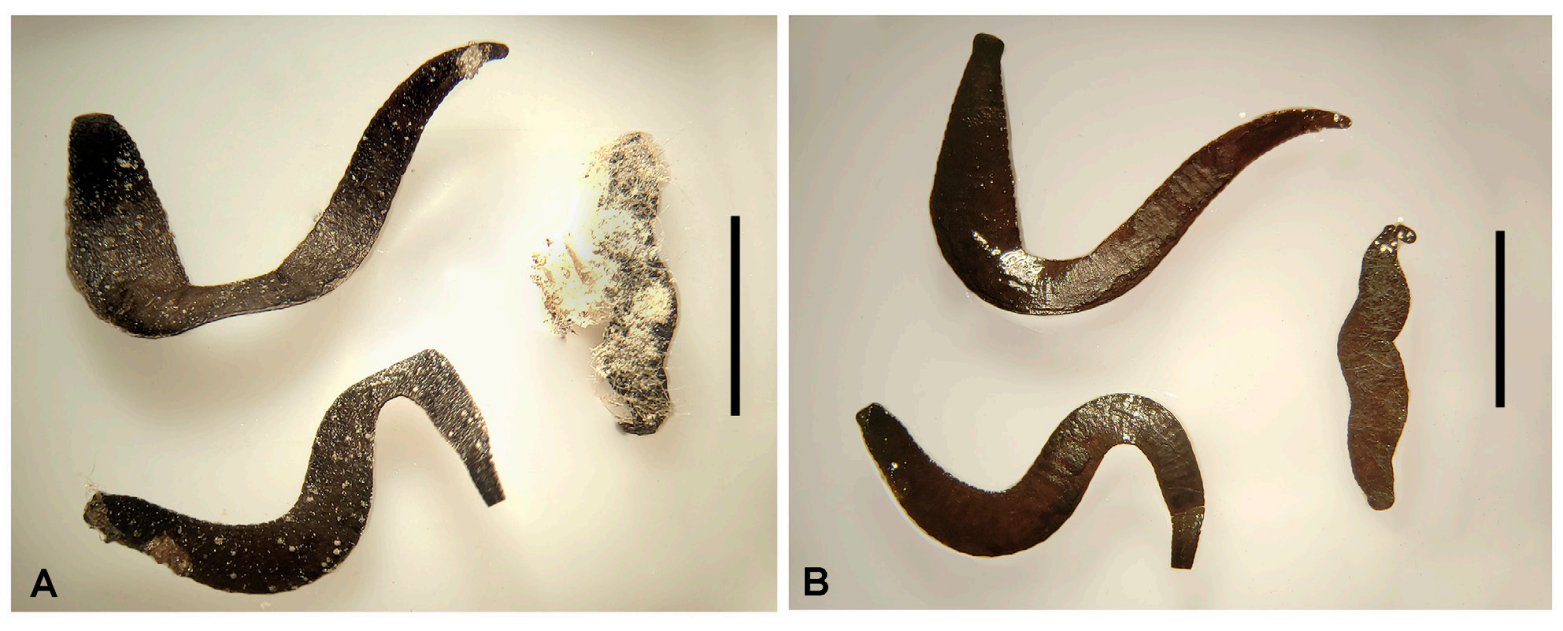
Specimens of Annelida before and after the rehydration technique. (A) Dried sample of Hirudinea (CHIOC 19792), before the rehydration technique; (B) Hirudinea (CHIOC 19792) after the rehydration technique. Bars A and B = 5 mm.

Class: Hirudinea (Figure 6A-6B).

Optimal rehydration time in distilled water: minimum three hours and maximum four hours.

Size: 0.6 to 7.5 cm.

Number of samples analyzed: 4.

## Discussion and Conclusion

Specimens stored in biological collections, if properly maintained, can last for hundreds of years, thus perpetuating the history of biodiversity (Papavero, 1994), as in the case of the helminths deposited in CHIOC. They can be used as a source of information for various fields of science, such as studies on biodiversity, biogeography, parasite-host relationship, conservation and dispersion of species, epidemiology and zoonosis. Currently, there are two reports on the use of TSP as a reagent for resurrecting preserved zoological specimens that have dried out through complete evaporation of their preserving liquid, including invertebrates such as leeches, cestodes, nematodes and acanthocephalans (van Cleave & Ross, 1947a, b).

van Cleave & Ross (1947a, b) used a rehydration technique consisting of use of commercial TSP, on some kinds of dried invertebrates. They tested TSP concentrations of 0.25% to 0.5% in distilled water and obtained highly satisfactory results from use of TSP on both small and larger specimens. Their results were not uniform for all kinds of dried specimens treated, but in every instance the recovered specimens showed restoration of general body form and revealed details of diagnostic characteristics that had been wholly unavailable in the dried specimens and in those treated with water or alcohol alone.

However, in the present study, the tests performed with TSP at the three dilutions (0.25%, 0.35% and 0.5%) did not prove that this is an efficient technique for recovering dried-out helminths. It was observed that when samples were subjected to 0.25% and 0.35% TSP, the samples showed tissue rupture after 50 and 40 minutes respectively, and still showed rigidity. With 0.50% TSP, tissue rupture was observed in all the specimens analyzed after 20 minutes. Furthermore, in the present study, it was found that dry specimens that were treated only with distilled water presented full recovery of their morphology (100% of the rehydrated samples).

van Cleave & Ross (1947a, p. 318) did not estimate the length of time for which the stored material had been desiccated; they just described this as “[...] many years [...]”. Here, we analyzed material stored in CHIOC that had been desiccated for at least 50 years. These were samples of dried invertebrates, mainly helminths, in a highly fragile state. Therefore, the ineffectiveness of TSP seen in the present study can be explained by the high degree of desiccation of the CHIOC specimens, which often were brittle, with presence of formalin crystals and fungi. The specimen restoration technique using only distilled water proved to be quite efficient for recovery of dried-out helminths in our study. This can be contrasted with the results obtained through recovery of specimens using TSP in comparison with the results from treatment with water or alcohol alone, reported by van Cleave & Ross (1947a).

Here, we presented the results from recovery of dried-out specimens deposited in CHIOC through rehydration using distilled water alone. This technique was developed with the objective of recovering the specimens’ malleability and flexibility, to enable morphological evaluation for taxonomic studies. Our results help to solve a big problem, in that many of the specimens deposited in collections may never be collected again because they are rare specimens of great scientific importance. This rehydration technique, together with constant maintenance of these collections, contributes to good functioning of these collections and to knowledge of and studies on the species therein deposited. Therefore, this intervention forms an important instrument for recovery of the specimens deposited in our historical collection, so that it remains a very important testimony to Brazilian biodiversity.
